# Mesenchymal Stem Cells With Cancer-Associated Fibroblast-Like Phenotype Stimulate SDF-1/CXCR4 Axis to Enhance the Growth and Invasion of B-Cell Acute Lymphoblastic Leukemia Cells Through Cell-to-Cell Communication

**DOI:** 10.3389/fcell.2021.708513

**Published:** 2021-10-18

**Authors:** Chengyun Pan, Qin Fang, Ping Liu, Dan Ma, Shuyun Cao, Luxin Zhang, Qingzhen Chen, Tianzhen Hu, Jishi Wang

**Affiliations:** ^1^School of Basic Medical Sciences, Guizhou Medical University, Guiyang, China; ^2^Department of Haematology, Affiliated Hospital of Guizhou Medical University, Guiyang, China; ^3^Hematological Institute of Guizhou Province, Guiyang, China; ^4^Department of Pharmacy, Affiliated Hospital of Guizhou Medical University, Guiyang, China; ^5^Guizhou Province Hematopoietic Stem Cell Transplantation Centre and Key Laboratory of Hematological Disease Diagnostic and Treatment Centre, Guiyang, China; ^6^National Clinical Research Center for Hematologic Diseases, The First Affiliated Hospital of Soochow University, Suzhou, China

**Keywords:** acute lymphoblastic leukemia, mesenchymal stem cells, cell–cell communication, cancer-associated fibroblast like phenotype, invasion

## Abstract

**Background:** Bone marrow mesenchymal stem cells (BM-MSCs) are the stromal cells in the leukemia microenvironment, and can obtain cancer-associated fibroblast (CAF)-like phenotype under certain conditions to further promote leukemia progression. However, the mechanism of MSCs with CAF-like phenotype interacting with leukemia cells in B-cell acute lymphoblastic leukemia (B-ALL) and promoting the progression of B-ALL remains unclear.

**Methods:** Mesenchymal stem cells with CAF-like phenotype were obtained by treating MSCs with recombinant human transforming growth factor-β (rhTGF-β), hereafter referred to as TGF-β conditioned MSCs. *In vivo* mouse model experiments, *in vitro* transwell chamber experiments, three-dimensional (3D) cell culture models, lentiviral transfection and other experimental methods were used to investigate the possible mechanism of the interaction between TGF-β conditioned MSCs and leukemia cells in promoting the growth, migration and invasion of B-ALL cells.

**Results:** Compared with untreated MSCs, TGF-β conditioned MSCs significantly promoted the growth and proliferation of leukemia cells in mice, and increased the expression of CXCR4 in tumor tissues. *In vitro* cell experiments, TGF-β conditioned MSCs obviously promoted the migration and invasion of Nalm-6/RS4;11 cells, which were effectively blocked by the CXCR4 inhibitor AMD3100, thereby inhibiting the secretion of MMP-9 in TGF-β conditioned MSCs and inhibiting the activation of the PI3K/AKT signaling pathway in leukemia cells. Further, findings were made that the interaction between TGF-β conditioned MSCs and leukemia cells were mediated by the interaction between the integrin receptor α5β1 on the surface of leukemia cells and the increased expression of fibronectin on TGF-β conditioned MSCs. AMD3100 could weaken such effect by reducing the expression of integrin α5β1 on leukemia cells. Further regulation of integrin β1 could effectively interfere with the interaction between TGF-β conditioned MSCs and leukemia cells.

**Conclusion:** Mesenchymal stem cells with CAF-like phenotype could be a key factor in promoting the growth and invasion of B-ALL cells, and the SDF-1/CXCR4 axis might be a significant factor in mediating the communication of MSCs with CAF-like phenotype and leukemia cells. To prevent the progression of B-ALL cells, blocking the SDF-1/CXCR4 axis with AMD3100 or targeting integrin β1 might be a potential therapeutic strategy.

## Introduction

Adult B-cell acute lymphoblastic leukemia (B-ALL) is a common malignant tumor in the hematological system. Blast cells accumulate in the bone marrow and inhibit normal hematopoiesis, and can also invade the central nervous system, lymph nodes, gonads and other tissues. Although studies have shown that B-ALL patients have an 80–90% chance of complete remission after receiving the first induction chemotherapy, the occurrence of resistance, recurrence and extramedullary infiltration significantly limits the long-term survival of partial patients (Hefazi and Litzow, 2018; DeAngelo et al., 2020). Thus, for prolonging the long-term survival of B-ALL patients, finding more effective interventions to improve the outcome of B-ALL and reduce leukemia infiltration is essential.

The bone marrow hematopoietic microenvironment is critical for leukemia cells to survive and proliferate. In recent years, the tumor microenvironment (TME) attracted widespread attention from scholars, and the role thereof has shifted from being a bystander and facilitator of tumors to having an indispensable and decisive role (Kotecha and Cheung, 2019; Dander et al., 2021). Through the extensive and multi-layered cross-talk between the tumor and stromal cells, the TME adapts to support tumor survival, growth and metastasis (Sánchez-Aguilera and Méndez-Ferrer, 2017; Delahaye et al., 2020). In the traditional treatment of leukemia, the poor curative effect can be partly attributed to the leukemia cells being protected by the surrounding stromal microenvironment, which provides a natural shelter for leukemia cells to avoid being killed by chemotherapeutics. The stromal microenvironment also provides time and conditions that guarantee infiltration of leukemia cells, and becomes a possible source of leukemia recurrence and progression (Chiarini et al., 2016; Shafat et al., 2017).

Cancer-associated fibroblasts (CAFs) are activated fibroblasts in the TME. An increasing amount of studies have indicated that CAFs have a causal effect on tumors from the early stage of the disease, and that the number of CAFs is closely related to tumor progression and poor prognosis (Jung et al., 2016; Alexander and Cukierman, 2020; Joshi et al., 2021). Over the years, the protective role of CAFs in several hematological malignancies has been gradually revealed (Frassanito et al., 2016; Zhai et al., 2016; Burt et al., 2019). Notably, in a previous *in vitro* study of the present authors, findings were made that bone marrow mesenchymal stem cells (BM-MSCs) could acquire CAF-like phenotype in the B-ALL microenvironment, and the TGF-β was a key factor for MSCs to obtain the CAF-like phenotype, with the latter potentially being a significant component in promoting the migration and invasion of B-ALL cells (Pan et al., 2020). However, there are a number of issues that remain unclear, such as how the MSCs with CAF-like phenotype interact with B-ALL cells and how the progression of B-ALL is promoted.

Chemokine receptor 4 (CXCR4) is a G protein-coupled receptor with seven transmembrane structures. CXCR4 is expressed on the surface of many tumor cells and in the cytoplasm, and the ligand stromal cell-derived factor 1 (SDF-1) thereof is a significant α-type chemokine, which is mainly produced by stromal cells. The specific binding of SDF-1 and CXCR4 form the SDF-1/CXCR4 biological axis, which is a key signal axis that mediates the interaction between tumor cells and stromal cells, and is highly related to cell adhesion, migration and invasion (de Lourdes Perim et al., 2015; Benedicto et al., 2018). However, whether the SDF-1/CXCR4 axis mediates the interaction between MSCs with CAF-like phenotype and leukemia cells in B-ALL is unclear.

In the present study, *in vivo* experiments were performed to verify whether MSCs with CAF-like phenotype are significant matrix components in promoting the progression of B-ALL. Further, investigations were conducted into whether the SDF-1/CXCR4 signal axis is a key axis that mediates the interaction between MSCs with CAF-like phenotype and ALL cells to promote the growth and invasion of leukemia cells, and also into the underlying downstream molecular mechanism of the cross-talk between MSCs with CAF-like phenotype and B-ALL cells through *in vitro* experiments and the three-dimensional (3D) cell culture model.

## Materials and Methods

### Cell Cultures

#### Acute Lymphoblastic Leukemia Cells and Normal Controls

The human ALL cell lines Nalm-6 and RS4;11 were obtained from the Laboratory of Haematopoietic Stem Cell Transplantation Center of Guizhou Province (Guiyang, China). The primary B-ALL cells were collected from the bone marrow mononuclear cells of three newly diagnosed CD19^+^ B-ALL patients who did not receive any treatment, and the bone marrow mononuclear cells of normal controls were obtained from the bone marrow of three healthy hematopoietic stem cell transplant donors. All patients and donors provided informed consent for participation. The cells were cultured in an RPMI-1640 medium supplemented with 10% fetal bovine serum, 100 units/mL penicillin and 100 mg/mL streptomycin at 37°C and 5% CO_2_ conditions.

#### Mesenchymal Stem Cells and Mesenchymal Stem Cells With Cancer-Associated Fibroblast-Like Phenotype

For collection of BM-MSCs, bone marrow aspirates were obtained with informed consent from a total of 26 primary B-ALL patients to complete the experiments in this study. And for each sample, the ficoll gradient centrifugation was conducted for BM-MSCs separation and cells at passages 2–4 were selected for further experiments. The complete medium of adult BM-MSCs (Saiye, Shanghai, China) was used for the cultivation of MSCs. MSCs with CAF-like phenotype were obtained by adding recombinant human transforming growth factor-β (rhTGF-β) (10 ng/ml) to stimulate MSCs for 48–72 h (Pan et al., 2020), which referred to as TGF-β conditioned MSCs.

#### Co-culture System

For co-culture of the two-dimensional (2D) cells, BM-MSCs (1 × 10^5^/ml) were initially plated for 24 h in the culture plate, and then ALL cells were added at a 4:1 ratio to form a co-cultivation system. Nalm-6/RS4;11 cells were separated from the BM-MSCs monolayer through careful pipetting. For the 3D cell culture, MSCs/TGF-β conditioned MSCs (1 × 10^5^/ml) were first inoculated on the 3D Insert^TM^ polystyrene (PS) scaffold (3D Biotek, Beijing Qunxiaokeyuan Biotechnology Co., Ltd., Beijing, China) in a 24-well plate. The method involved resuspending the cells in 60 μl medium and then dropping the cell suspension onto the center of the 3D Insert^TM^ PS scaffold. After the plate was gently placed in the incubator for 3 h, the 24-well plate was removed from the incubator, 940 μl culture medium was added, and culture of the cells was continued on the scaffold at 37°C and 5% CO_2_ conditions. For the 3D co-culture system, after inoculating MSCs/TGF-β conditioned MSCs in the 3D Insert^TM^ PS scaffold for 24 h, leukemia cells 4 × 10^5^/ml were inoculated for co-culture.

### Reagents and Antibodies

RhTGF-β was purchased from Solibao Biotechnology Co., Ltd. (Beijing, China) and was formulated in enzyme-free water. AMD3100 was provided by MedChemExpress (Shanghai, China), formulated in absolute ethanol at a stock concentration of 100 mM, and diluted to a working fluid of 10 mM with RPMI-1640. The PI3K, AKT, phospho-AKT, ERK and phospho-ERK antibodies were obtained from Cell Signaling Technology (United States), the phospho-PI3K was purchased from Affinity Biosciences (OH, United States). The fibronectin (FN), collagen type I (COL-I), collagen type III (COL-III), integrin α5 (ITGA5) and integrin β1 (ITGB1) antibodies were obtained from Proteintech Group, Inc (Wuhan, China), the α-smooth muscle actin (α-SMA) and fibroblast activation protein (FAP) antibodies were purchased from Abcam (United Kingdom), while CXCR4 antibody was purchased from Solibao Biotechnology Co., Ltd. (Beijing, China). The secondary antibody used for western blot came from Medical Discovery Leader Corp. (Beijing, China). The fluorescent secondary antibody of Alexa Fluor^TM^ 488 donkey anti-rabbit IgG (H + L) was provided by invitrogen of Thermo Fisher Scientific (United States), and the Goat Anti-Mouse IgG (H + L), CoraLite594 conjugate secondary antibody was purchased from Proteintech Group, Inc (Wuhan, China). Western Blot Electrochemiluminescence (ECL) Reagent was obtained from 7Sea Pharmatech Co., Ltd. (Shanghai, China).

### Cell Migration and Invasion

For cell migration (transwell chamber) and invasion (transwell chamber covered with matrigel) analysis, MSCs and TGF-β conditioned MSCs (1 × 10^5^/mL) were added into the lower transwell chamber for overnight incubation, and Nalm-6/RS4;11 cell lines (4 × 10^5^/mL) were added into the upper chamber (8.0 μm pore size, Corning Incorporated, Costar). After incubating for 24 h, the migration and invasion numbers of leukemia cells in the lower chamber were measured under an inverted microscope, 200 × pictures were taken, and five random fields of view were selected to count the migration and invasion cell numbers.

### Lentiviral Transduction

Human ITGB1-silencing RNA (si-ITGB1), Human ITGB1 overexpress clone lentiviral particle (LV-ITGB1) and Human CXCR4 overexpress clone lentiviral particle (LV-CXCR4) was purchased from Genechem Co., Ltd. (Shanghai, China). As for the information of lentiviral vectors, *Escherichia coli* strain DH5α was used to amplify lentiviral vectors and auxiliary packaging vector plasmids. There were three plasmids involved in virus packaging, which included the tool vector plasmid carrying the target gene or the target sequence; the virus packaging helper plasmid (Helper 1.0) and the virus packaging helper plasmid (Helper 2.0). Transfection of si-ITGB1/LV-ITGB1/LV-CXCR4 was performed in accordance with the instructions of the manufacturer. Nalm-6/RS4;11 cells transfected with empty vector (EV) were applied as controls. After expansion and maintenance in an RPMI-1640 medium supplemented with 10% FBS for 5 days, stable Nalm-6/RS4;11 cells expressing si-ITGB1/LV-ITGB1/LV-CXCR4 were selected by using puromycin (1 μg/ml for Nalm-6 cell lines and 2 μg/ml for RS4;11 cell lines).

### Flow Cytometry

After being treated with different doses of AMD3100 for 24 h, leukemia cells were collected for apoptosis detection. To detect apoptosis of leukemia, cells were stained with Annexin-V for 15 min and propidium iodide (PI) for 5 min in accordance with the recommended protocol (7Sea Pharmatech Co., Ltd, Shanghai, China). The data were analyzed by using a flow cytometer (BD Biosciences, San Jose, CA, United States).

### Quantitative Real-Time Polymerase Chain Reaction

Total RNA was extracted using the RNeasy kit (Qiagen, Hilden, Germany) and reversely transcribed to cDNA (the Omniscript reverse transcription kit, Qiagen). Subsequently, the primers and iQ SYBR Green Supermix (Bio-Rad, Hercules, CA, United States) were used to analyze cDNA by Quantitative real-time polymerase chain reaction (RT-PCR) in accordance with the protocol of the manufacturer. The relative expression of the target gene was detected by the comparative CT method (2^–Δ*CT*^), utilizing β-actin as the internal control. The following human primers (Generay Biotech Co. Ltd, Shanghai, China) were used in the present study:

β-actin (F), CTACCTCATGAAGATCCTCACCGA
β-actin (R), TTCTCCTTAATGTCACGCACGATT
ITGB1 (F), CGTGCAAATCCCACAAC
ITGB1 (R), CCCTGGCATGAATTACAAC
CXCR4 (F), ACTACACCGAGGAAATGGGCT
CXCR4(R), CCCACAATGCCAGTTAAGAAGA

### Western Blotting and Enzyme-Linked Immunosorbent Assay

Leukemia cells and stromal cells were harvested and lysed using RIPA lysis buffer (Beyotime, Shanghai, China) containing 1% phenylmethylsulfonyl fluoride (PMSF) (Beyotime, Shanghai, China). 10–30 μg protein was fractionated by SDS-PAGE and transferred onto the PVDF membrane. Subsequently, the membrane was blocked with 5% skimmed milk for 1−2 h and then incubated with the primary antibody at 4°C overnight. The diluted concentration of the target protein antibodies (PI3K, phospho-PI3K, AKT, phospho-AKT, ERK, phospho-ERK, FN, COL-I, COL-III, ITGA5 and ITGB1 antibodies) was 1:1000, and the diluted concentration of the β-actin antibodies was 1:2000. After being washed with Tris-Buffered Saline and Tween 20 (TBST), the membrane was incubated with secondary antibody (1:2000) for 1 h, and followed by the ECL reagent to detect the protein expression. Finally, the obtained bands were analyzed by the Image J software for gray value analysis, with β-actin as an internal reference. Enzyme-linked immunosorbent assay (ELISA) for cytokines was performed in accordance with the instructions of the manufacturer (Beijing 4A Biotech Co., Ltd., Beijing, China).

### Immunofluorescence Staining and Immunohistochemical Staining

Suspended leukemia cells and adherent stromal cells were fixed with 4% paraformaldehyde for 30 min or at 4°C overnight. After washing the cells in the slide with PBS, the cells were permeabilized with 0.1% Triton-X100 for 10–15 min, then incubated with 5% fresh goat serum for 1 h, probed with specific primary anti-FN (1:100), anti-COL-III (1:100), anti-COL-I (1:100), anti-ITGA5 (1:50), and anti-ITGB1 (1:50) antibodies at 4°C overnight, followed by incubation with fluorescence-labeled secondary antibodies (1:200) for 1 h in the dark. Finally, the nuclei were counterstained with DAPI for 5 min, and the fluorescence images were captured by the fluorescence microscope. Immunohistochemical (IHC) staining was conducted according to specific instructions, and the dilution concentrations of the primary and secondary antibodies were consistent with immunofluorescence (IF) staining. A staining index was calculated by multiplying the degree of staining of positive cells by the proportion of positive cells. The degree of staining of positive cells was scored as follows: negative, 0; weakly positive, 1; moderately positive, 2; strong positive, 3. Proportion of positive cells was defined as follows: less than 5%, 0; 5–25%, 1; 26–50%, 2; 51–75%, 3; greater than 75%, 4.

### Cell Adhesion

Stromal cells (1 × 10^5^ cells/ml) were plated on a 24-well plate and cultured for 24 h. In accordance with the instructions of the manufacturer, Nalm-6/RS4;11 cells were stained with Calcein-AM fluorescent probe (Beyotime Biotechnology Co., Ltd., Shanghai, China) for 30 min to label as green fluorescence, then washed with PBS, and resuspended in a fresh medium to make the cell density 4 × 10^5^/ml. Subsequently, 500 μl leukemia cells suspension was added to each 24-well plate of stromal cells, the suspended supernatant was gently aspirated after co-culturing for 24 h, and the adhered leukemia cells labeled with green fluorescence were checked under a fluorescent microscope. For quantitative analysis in each group, 5 random fields of view were taken for the adhered cell count, and then the adhesion rate of leukemia cells was calculated by dividing the number of adhered cells of the experimental group by the number of adhered cells of the control group, and the values of control group were normalized to 100%.

### Electron Microscopy

For electron microscopy, cells grown on the 3D Insert^TM^ PS scaffolds were fixed by 2.5% glutaraldehyde for 24 h and were dehydrated in a graded series of ethanol (50, 70, 90, and 100%) for 15 min interval in each gradient, and then used for electron microscopic study with standard methods.

### Xenografted Tumor Model

Mice xenograft assays were approved by the Animal Care Welfare Committee of Guizhou Medical University. The animal studies utilized non-obese diabetes/severe combined immunodeficiency (NOD/SCID) male mice aged 4–6 weeks, which were subcutaneously injected with Nalm-6 cells, or a mixture of Nalm-6 cells and MSCs, or a mixture of Nalm-6 cells and TGF-β conditioned MSCs (1 × 10^6^ MSCs or TGF-β conditioned MSCs mixed with 4 × 10^6^ Nalm-6 cells) were injected subcutaneously into the left chest of each mouse. The mice were observed for 40 days after being injected with leukemia cells. Tumor growth was monitored at 5–7 day intervals by measuring the length (L) and width (W) of the tumor with calipers and calculating the tumor volume based on the following formula: volume = 0.5 LW^2^. The mice were sacrificed, and the tumors were harvested and measured.

### Statistical Analysis

Data were analyzed by using SPSS 19.0 software and were evaluated with independent Student’s *t*-test for analysis between two groups, while one way ANOVA was used for analysis among multiple groups with homogeneity of variance. Non-parametric test (the Kruskal–Wallis test) was used for animal experimental data that did not passed the normality test. Data were showed as mean ± standard deviation (SD), and P-values were assigned as follows (^∗^*P* < 0.05; ^∗∗^*P* < 0.01; ^∗∗∗^*P* < 0.001). For the comparison between multiple groups, after the *Post hoc* test, the significance level of *P*-value was corrected by Bonferroni method, the correction formula was: 0.05/number of pairwise comparisons.

## Results

### Identification of Bone Marrow Mesenchymal Stem Cells and Mesenchymal Stem Cells With Cancer-Associated Fibroblast-Like Phenotype

In the present study, BM-MSCs were derived from bone marrow mononuclear cells of patients with B-ALL. The phenotypic characteristics, adipogenic and osteogenic differentiation functions thereof have been verified in previous research by the present authors (Pan et al., 2020). MSCs with CAF-like phenotype were obtained from BM-MSCs of the same B-ALL patient after stimulation with rhTGF-β for 48–72 h (TGF-β conditioned MSCs). The morphological characteristics and the expression level of biomarkers were used to identify and distinguish MSCs and MSCs with CAF-like phenotype (Pan et al., 2020). In the present study, the results of flow cytometry revealed that TGF-β conditioned MSCs highly expressed CD90, CD105 and CD44, but did not express CD34, CD45, and CD19 (Supplementary Figure S1A), which was consistent with the immunophenotype of MSCs (Pan et al., 2020). In terms of multi-directional differentiation functions, TGF-β conditioned MSCs had similar osteogenic differentiation ability compared with MSCs, but the adipogenic differentiation function was weakened (Supplementary Figures S1B,C).

### Mesenchymal Stem Cells With Cancer-Associated Fibroblast-Like Phenotype Interacted With Acute Lymphoblastic Leukemia Cells to Promote Leukemia Cell Growth, Proliferation and Increase the Expression of CXCR4 *in vivo*

**FIGURE 1:**
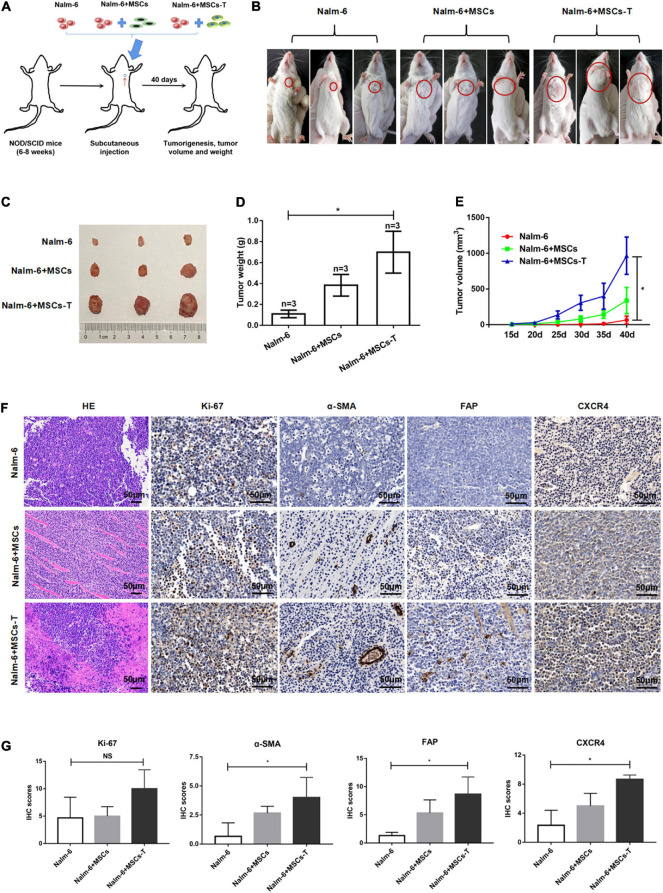
Mesenchymal stem cells with CAF-like phenotype promoted the growth and proliferation of ALL cells *in vivo*. **(A)** Model of mouse subcutaneous tumor formation. **(B,C)** The size of subcutaneous tumors in the Nalm-6 injection group (*n* = 3), Nalm-6 + MSCs injection group (*n* = 3), and Nalm-6 + TGF-β conditioned MSCs (MSCs-T) injection group (*n* = 3) after 40 days of growth. **(D)** Average tumor weight in each group was calculated at 40 days after injection. Error bars depict the mean ± SD. *P < 0.05. **(E)** Average tumor volume in each group, the difference of tumor volume between the three groups was evaluated at 40 days after injection. Error bars depict the mean ± SD. *P < 0.05. **(F)** HE staining (200×) of tumor tissue and the expression of Ki-67, α-SMA, FAP, and CXCR4 (400×) in xenotransplanted tumors with IHC (scale bars, 50 μm). **(G)** The IHC scores of Ki-67, α-SMA, FAP and CXCR4 in each group. NS, no significant statistical difference.

Here, the effects of MSCs with CAF-like phenotype on the growth and proliferation of ALL cells were first explored through *in vivo* experiments. To evaluate the contribution of MSCs with CAF-like phenotype to tumor progression *in vivo*, a human tumor xenograft model was developed that had stromal compartments in the engrafted tumors. MSCs or TGF-β conditioned MSCs were mixed with Nalm-6 cells at a ratio of 1:4 and the mixture was subcutaneously injected into the left chest of immunodeficiency mice (Figure 1A). The tumor formation was monitored. As shown in Figures 1B–E, compared with Nalm-6 cells mixed with MSCs (*n* = 3) or Nalm-6 cells alone (*n* = 3), tumors of greater volume and weight were generated by Nalm-6 cells co-injected with TGF-β conditioned MSCs (*n* = 3). The stromal fibroblasts phenotypes were detected by IHC using antibodies specific for human α-SMA and FAP, which are significant markers of CAFs. The TGF-β conditioned MSCs group were positive for α-SMA and FAP expression, and the MSCs group were moderately positive for α-SMA and FAP expression (Figures 1F,G). Additionally, the proliferation of antigen Ki-67 was detected, with the results revealing that TGF-β conditioned MSCs combined with ALL cells injection seemed to increase the expression of Ki-67, but there was no statistical difference between the groups (Figures 1F,G). Further, the results of HE staining revealed that the matrix components of the TGF-β conditioned MSCs and Nalm-6 cells co-injection group had a higher tendency than those in the MSCs and Nalm-6 co-injection group and the Nalm-6 alone injection group (Figure 1F), suggesting that TGF-β conditioned MSCs might produce more matrix components to support the growth and proliferation of leukemia cells in tumor tissues.

Since the SDF-1/CXCR4 axis was considered as a significant signal axis in mediating the communication between tumor cells and stromal cells, the expression of CXCR4 was also detected in tumor tissue by using the IHC method. As shown in Figures 1F,G, the expression of CXCR4 in the TGF-β conditioned MSCs and Nalm-6 cells co-injection group tended to be higher than that in the MSCs and Nalm-6 cells co-injection group and the Nalm-6 cells alone injection group, indicating that the increased expression of CXCR4 might be closely related to the interaction between MSCs with CAF-like phenotype and leukemia cells.

### Mesenchymal Stem Cells With Cancer-Associated Fibroblast-Like Phenotype Promote the Migration and Invasion of Acute Lymphoblastic Leukemia Cells *in vitro* via SDF-1/CXCR4 Signaling

**FIGURE 2:**
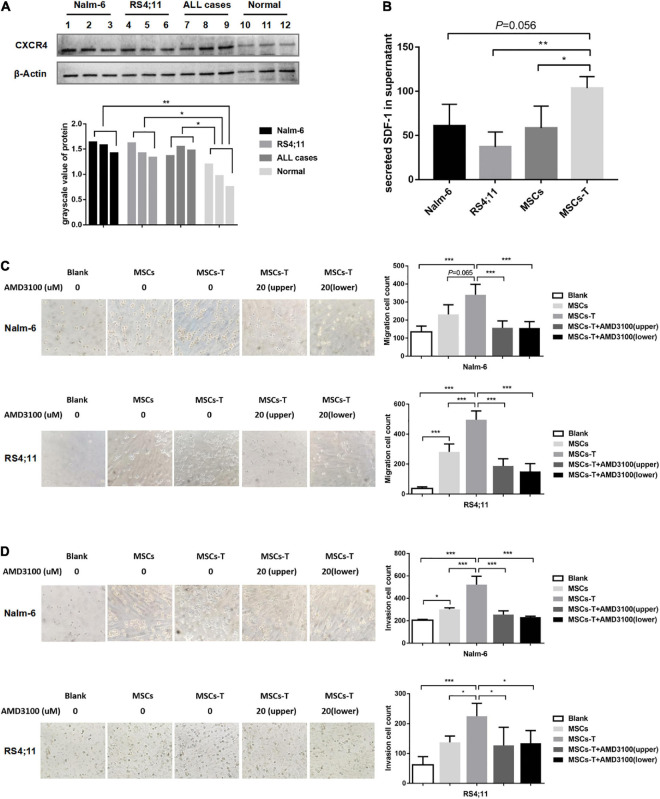
Mesenchymal stem cells with CAF-like phenotype promoted the migration and invasion of ALL cells *in vitro* via SDF-1/CXCR4 signaling. **(A)** The expression of CXCR4 in Nalm-6 cell lines, RS4;11 cell lines, ALL cases (*n* = 3) and bone marrow mononuclear cells of normal controls (*n* = 3) by western blot. **(B)** ELISA showing the supernatant expression of SDF-1 (pg/ml) in the medium of Nalm-6 cell lines, RS4;11 cell lines, MSCs and TGF-β conditioned MSCs. **(C,D)** Transwell chamber test to detect the number of Nalm-6 and RS4;11 cells migration and invasion in blank, MSCs, TGF-β conditioned MSCs, TGF-β conditioned MSCs + AMD3100 (20 μM, upper chambers) and TGF-β conditioned MSCs + AMD3100 (20 μM, lower chambers) group after 24 h of cells incubation. Each value indicates the mean ± SD of three or more independent experiments (Figures 2B–D) or three independent samples (Figure 2A). In each independent repeated experiment, MSCs was isolated and cultured from a B-ALL patient, and stimulated with rhTGF-β to obtain the CAF-like phenotype, then the latter were compared with the untreated MSCs from the same patient to ensure that the experiment was comparable. *P < 0.05, **P < 0.01, ***P < 0.001.

The malignant proliferation of cells is a significant basis for tumor cell migration and invasion. Through the *in vivo* experiments in the present study, an investigation was conducted into whether MSCs with CAF-like phenotype could promote the migration and invasion of ALL cells through the SDF-1/CXCR4 axis. First, the expression of CXCR4 was detected in leukemia cells and normal controls (*n* = 3), from which CXCR4 was found to be highly expressed to varying degrees in Nalm-6, RS4;11 and primary B-ALL cells (*n* = 3) (*P* < 0.05; Figure 2A). The ELISA method was used to detect the expression of SDF-1 in the supernatant of Nalm-6, RS4;11, MSCs and TGF-β conditioned MSCs. The results revealed that the expression of SDF-1 in TGF-β conditioned MSCs had a higher tendency than the expression in other groups (Figure 2B). In previous research, the present authors found that CXCR4 was also expressed in stromal cells (Pan et al., 2020), and the increase of SDF-1 in CAFs has been reported in previous study (Izumi et al., 2016). An assumption was made that the increased SDF-1 secreted by TGF-β conditioned MSCs combines with the receptor CXCR4 in the TME to stimulate the activation of the SDF-1/CXCR4 signal axis, thereby promoting the interaction between MSCs with CAF-like phenotype and B-ALL cells, which promotes the migration and invasion process of B-ALL cells. Therefore, in the following experiment, AMD3100 was used, a specific CXCR4 inhibitor, with an appropriate dosage to block the SDF-1/CXCR4 signal axis (Supplementary Figure S2). Moreover, transwell chambers were used to detect the effects of blank, MSCs, TGF-β conditioned MSCs and TGF-β conditioned MSCs + AMD3100 groups on the migration and invasion of Nalm-6/RS4;11 cells. The results revealed that, compared with the MSCs and blank groups, TGF-β conditioned MSCs had a more obvious tendency to promote the migration and invasion of Nalm-6/RS4;11 cells (Figures 2C,D). However, when AMD3100 was added to the upper or lower chamber of the transwell to block the SDF-1/CXCR4 signal axis, the promotion effect of TGF-β conditioned MSCs was weakened (*P* < 0.05; Figures 2C,D). To further verify the role of SDF-1/CXCR4 axis in the interaction between TGF-β conditioned MSCs and ALL cells, CXCR4 was overexpressed in Nalm-6/RS4;11 cells through lentiviral transfection, the successful up-regulation of CXCR4 was verified through RT-PCR and Western blot methods (Supplementary Figures S3A,B), and the effect of TGF-β conditioned MSCs on the migration and invasion of leukemia cells was further tested. The results revealed that compared with the leukemia cells in the control group and EV group, up-regulating CXCR4 in leukemia cells obviously enhanced the promotion effect of TGF-β conditioned MSCs on the migration and invasion of Nalm-6/RS4;11 cells (*P* < 0.05; Supplementary Figures S3C,D). Suggesting that the SDF-1/CXCR4 signal axis might be a significant factor in the process of MSCs with CAF-like phenotype promoting the migration and invasion of leukemia cells.

### AMD3100 Inhibits the Secretion of MMP-9 in Mesenchymal Stem Cells With Cancer-Associated Fibroblast-Like Phenotype, and Inhibits the Activation of PI3K/AKT Signaling Pathway in Acute Lymphoblastic Leukemia Cells

**FIGURE 3:**
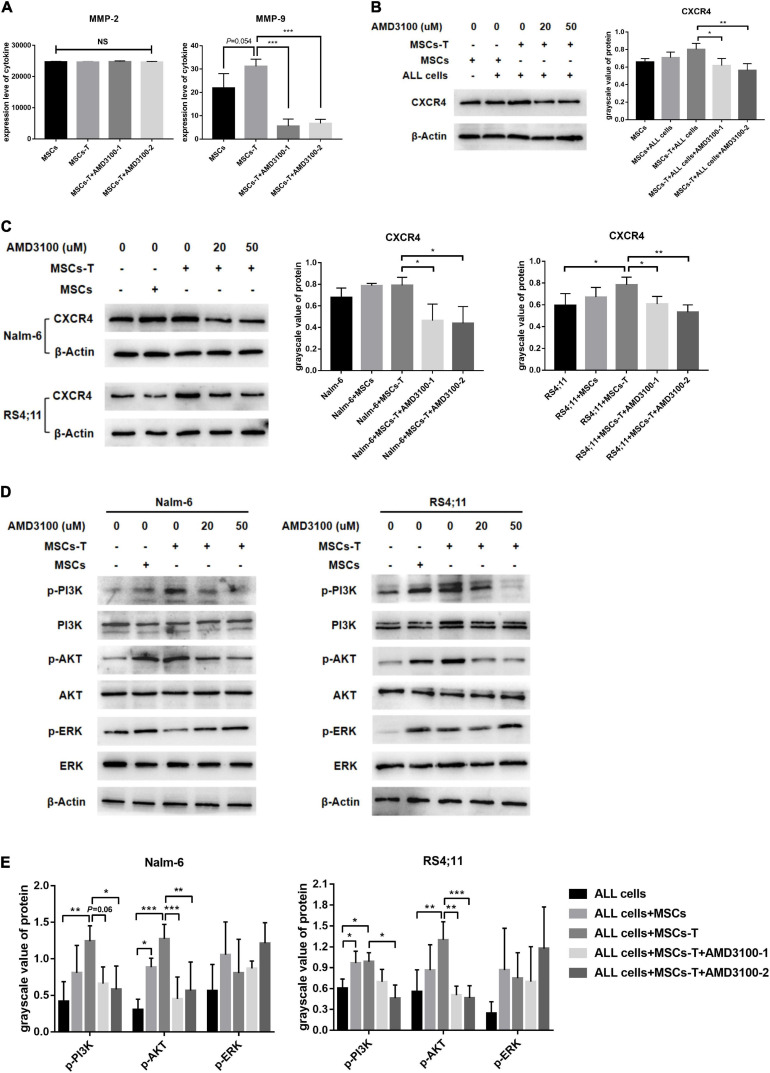
AMD3100 inhibited the secretion of MMP-9 in MSCs with CAF-like phenotype, and inhibited the activation of PI3K/AKT signaling pathway in ALL cells. **(A)** ELISA showing the supernatant expression of MMP-2 (pg/ml) and MMP-9 (pg/ml) in the medium of MSCs, TGF-β conditioned MSCs, TGF-β conditioned MSCs + AMD3100-1 (20 μM) and TGF-β conditioned MSCs + AMD3100-2 (50 μM). **(B)** The expression of CXCR4 in the stromal cells of MSCs group, MSCs + ALL cells group, TGF-β conditioned MSCs + ALL cells group, TGF-β conditioned MSCs + ALL cells + AMD3100-1 (20 μM) group and TGF-β conditioned MSCs + ALL cells + AMD3100-2 (50 μM) group by western blot after culturing 24 h. **(C)** The expression of CXCR4 in Nalm-6/RS4;11 cells from individual culture and co-culture with MSCs, TGF-β conditioned MSCs, TGF-β conditioned MSCs + AMD3100-1 (20 μM) and TGF-β conditioned MSCs + AMD3100-2 (50 μM) for 24 h by western blot. **(D)** The expression of p-PI3K, p-AKT, and p-ERK in Nalm-6/RS4;11 cells from individual culture and co-culture with MSCs, TGF-β conditioned MSCs, TGF-β conditioned MSCs + AMD3100-1 (20 μM) and TGF-β conditioned MSCs + AMD3100-2 (50 μM) for 24 h. **(E)** Gray value analysis of p-PI3K, p-AKT, and p-ERK signal pathways expressed in the above five groups, with the total protein corresponding to the phosphorylated protein as an internal control. Each value indicates the mean ± SD of three or more independent experiments. In each independent repeated experiment, MSCs was isolated and cultured from a B-ALL patient, and stimulated with rhTGF-β to obtain the CAF-like phenotype, then the latter were compared with the untreated MSCs from the same patient to ensure that the experiment was comparable. *P < 0.05, **P < 0.01, ***P < 0.001.

Mesenchymal stem cells with CAF-like phenotype could increase the migration and invasion of B-ALL cells thereof through the transwell system. This effects did not require direct cell–cell contact. Correlated with the aforementioned finding, conditioned media from MSCs, TGF-β conditioned MSCs and TGF-β conditioned MSCs + AMD3100 were analyzed by ELISA to detect the expression levels of matrix metalloproteinase (MMP) family members MMP-2 and MMP-9, which can promote tumor cells to invade surrounding tissues and metastasize to distant tissues in a variety of tumors (Fang et al., 2021; Yin et al., 2021). As shown in Figure 3A, the expression level of MMP-2 was not significantly different between MSCs and TGF-β conditioned MSCs, and there was no obvious change after AMD3100 treatment. However, for MMP-9, the results revealed that the conditioned media from TGF-β conditioned MSCs resulted in a certain degree of MMP-9 increase compared with the conditioned media from MSCs (*P* = 0.054). When TGF-β conditioned MSCs were treated with AMD3100, MMP-9 expression was reduced (*P* < 0.05; Figure 3A). Such findings suggested that MSCs with CAF-like phenotype might mediate the migration and invasion of ALL cells partially via paracrine MMP-9, and AMD3100 could inhibit such effects by reducing the secretion of MMP-9 in MSCs with CAF-like phenotype.

In addition, further observations were made on the possible changes of the related downstream signaling pathway in ALL cells during the interaction between MSCs with CAF-like phenotype and ALL cells, and after blocking the SDF-1/CXCR4 axis. Previous studies have reported that the specific binding of the SDF-1/CXCR4 axis can activate the phosphorylation of a variety of downstream pro-tumor survival signaling pathways (Xu et al., 2015). Among them, ERK and PI3K/AKT signals are significant in regulating the proliferation and migration of leukemia cells (Chang et al., 2020). Hence, the changes in the ERK and PI3K/AKT signaling pathways in B-ALL cells were detected. Here, the expression of CXCR4 in the co-culture system and the inhibitory effect of AMD3100 on CXCR4 were first detected. Results revealed that the expression of CXCR4 in leukemia cells in the TGF-β conditioned MSCs co-culture system had a tendency to increase compared with the ALL cells cultured alone, but had no statistical difference between the group in Nalm-6 cells, adding a certain dose of AMD3100 to the co-culture system could effectively inhibit the expression of CXCR4 in TGF-β conditioned MSCs and ALL cells (*P* < 0.05; Figures 3B,C). For the signaling pathways, the phosphorylated PI3K (p-PI3K) and phosphorylated AKT (p-AKT) were obviously activated in the co-culture system of TGF-β conditioned MSCs and Nalm-6/RS4;11 cells group compared with Nalm-6/RS4;11 cells culture alone, but there was no statistical difference compared with the co-culture system of MSCs and Nalm-6/RS4;11 cells group. Blocking the SDF-1/CXCR4 axis with AMD3100 could inhibit the expression level of p-PI3K and p-AKT, while the ERK signal did not change obviously under AMD3100 treatment (Figures 3D,E). Suggesting that the PI3K/AKT signaling pathway in leukemia cells might be activated to varying degrees when co-cultured with stromal cells, and AMD3100 might reduce the expression of p-PI3K and p-AKT on leukemia cells to a certain extent by interfering with the interaction between stromal cells and leukemia cells.

### AMD3100 Weakened the Interaction Between Mesenchymal Stem Cells With Cancer-Associated Fibroblast-Like Phenotype and Acute Lymphoblastic Leukemia Cells by Reducing the Adhesion of Leukemia Cells

**FIGURE 4:**
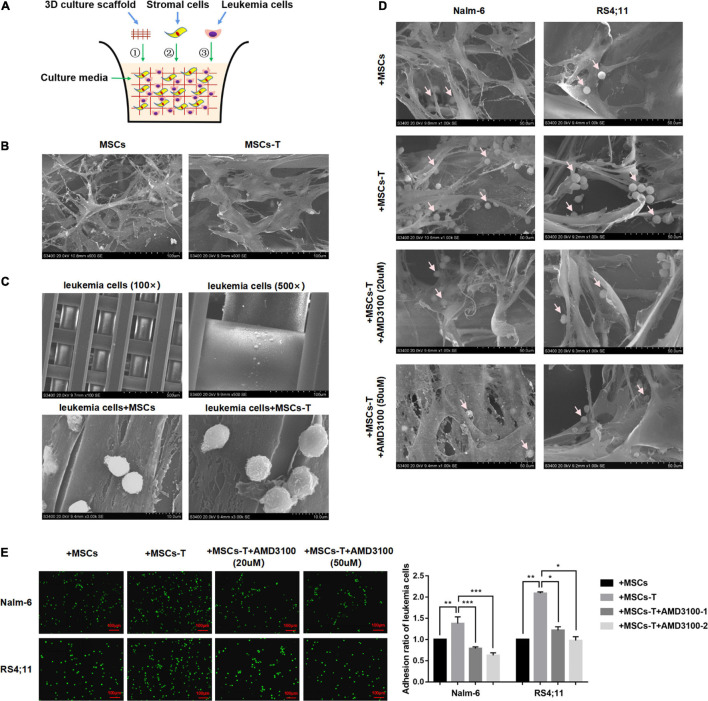
AMD3100 weakened the interaction between MSCs with CAF-like phenotype and ALL cells by reducing the adhesion of leukemia cells to MSCs with CAF-like phenotype. **(A)** The structure simulation diagram of the 3D cell culture model. **(B)** The cell morphology of MSCs and TGF-β conditioned MSCs when they cultured in a 3D scaffold (500×). **(C)** Representative pictures of leukemia cells culture alone (100× and 500×) or co-culture with MSCs/TGF-β conditioned MSCs (3000×) in the 3D scaffold. **(D)** The adhesion of leukemia cells to stromal cells when Nalm-6/RS4;11 cells were co-cultured with MSCs, TGF-β conditioned MSCs, TGF-β conditioned MSCs + AMD3100 (20 μM) and TGF-β conditioned MSCs + AMD3100 (50 μM) in a 3D scaffold for 24 h (1000×). The arrow in the figure refers to leukemia cells. **(E)** The adhesion of leukemia cells to stromal cells when Nalm-6/RS4;11 cells were co-cultured with MSCs, TGF-β conditioned MSCs, TGF-β conditioned MSCs + AMD3100 (20 μM) and TGF-β conditioned MSCs + AMD3100 (50 μM) for 24 h by using the fluorescence quantitative method (100×, scale bars, 100 μm). Each value indicates the mean ± SD of three or more independent experiments. In each independent repeated experiment, MSCs was isolated and cultured from a B-ALL patient, and stimulated with rhTGF-β to obtain the CAF-like phenotype, then the latter were compared with the untreated MSCs from the same patient to ensure that the experiment was comparable. *P < 0.05, **P < 0.01, ***P < 0.001.

Since the direct interaction between CAFs and tumor cells is another significant factor leading to tumor cell invasion and metastasis (Miyazaki et al., 2020; Zhang Y. et al., 2020). In the present *in vitro* cell experiments, considering that the traditional 2D cell culture cannot generalize the complex *in vivo* structure, a 3D cell culture model was adopted, which allows leukemia cells *in vitro* to grow in all directions, such that the appropriate spatial distribution and connection between cells can be formed. Here, a polystyrene scaffold material with optimized structure was selected to form a 3D Insert^TM^ PS scaffold model (Figure 4A), so as to more intuitively observe the interaction between MSCs with CAF-like phenotype and ALL cells and simulate the tumor microenvironment *in vivo*.

We first observed the morphological differences between MSCs and TGF-β conditioned MSCs during culture in the 3D Insert^TM^ PS scaffolds. As shown in Figure 4B, when seeded on the scaffold, MSCs/TGF-β conditioned MSCs could adhere tightly to the scaffold and grow in a spindle shape. Compared with MSCs, the TGF-β conditioned MSCs were obviously enlarged, and the cytoplasm contained various fiber filaments. However, leukemia cells cultured in the 3D Insert^TM^ PS scaffolds alone did not adhere to the scaffold unless they were co-cultured with MSCs/TGF-β conditioned MSCs (Figure 4C). In the co-culture system, several leukemia cells were hidden in the matrix fiber layer formed by the intersection of stromal cell fibers, and the number of leukemia cells attached to TGF-β conditioned MSCs seemed to be more than that of the co-culture system of MSCs, but decreased after AMD3100 was added to the co-culture system (Figure 4D). The fluorescent quantitative method was used to further detect the adherent leukemia cells, which were labeled with green fluorescence by Calcein-AM fluorescent probe, and then co-cultured with stromal cells for 24 h. After the suspension cells were removed, the adherent leukemia cells on stromal cells were observed by fluorescence microscope, and the adherent leukemia cells were counted. The results (*P* < 0.05; Figure 4E) were consistent with those of the 3D co-culture experiment. According to the aforementioned findings, the suggestion was that the direct co-culture of MSCs with CAF-like phenotype and B-ALL cells might increase the adhesion of ALL cells to stromal cells to promote direct interaction there between, AMD3100 could effectively reduce the occurrence of such interaction.

### Mesenchymal Stem Cells With Cancer-Associated Fibroblast-Like Phenotype Secrete Enhanced Extracellular Matrix Protein to Promote the Adhesion of Leukemia Cells, but AMD3100 Cannot Effectively Reverse the Increased Matrix Protein

**FIGURE 5:**
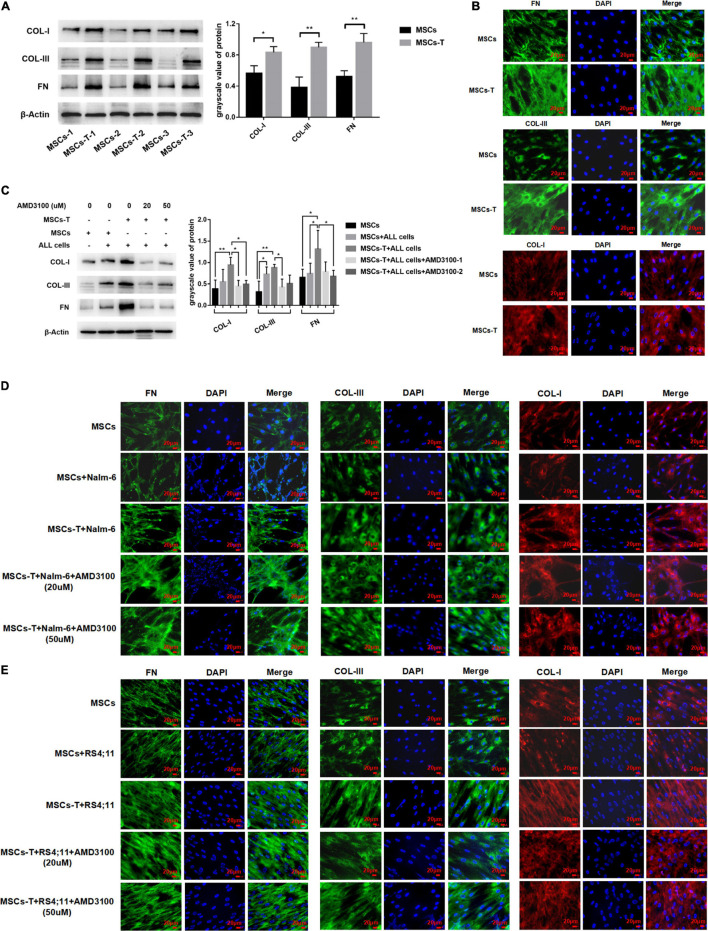
Mesenchymal stem cells with CAF-like phenotype secreted enhanced extracellular matrix protein to promote the adhesion of leukemia cells, but AMD3100 cannot effectively reverse the increased matrix protein. **(A)** The expression of Collagen-I (COL-I), Collagen-III (COL-I), and Fibronectin (FN) in MSCs (*n* = 3) and TGF-β conditioned MSCs (*n* = 3) by western blot. Each value indicates the mean ± SD. *P < 0.05, **P < 0.01. **(B)** The expression of FN, COL-III and COL-I in MSCs and TGF-β conditioned MSCs group by Immunofluorescence (400×, scale bars, 20 μm). **(C)** FN, COL-III, and COL-I in stromal cells of MSCs group, MSCs + ALL cells group, TGF-β conditioned MSCs + ALL cells group, TGF-β conditioned MSCs + ALL cells + AMD3100 (20 μM) group and TGF-β conditioned MSCs + ALL cells + AMD3100 (50 μM) group after culturing for 24 h by western blot. Each value indicates the mean ± SD of three or more independent experiments. In each independent repeated experiment, MSCs was isolated and cultured from a B-ALL patient, and stimulated with rhTGF-β to obtain the CAF-like phenotype, then the latter were compared with the untreated MSCs from the same patient to ensure that the experiment was comparable. *P < 0.05, **P < 0.01. **(D,E)** IF was used to detect the expression of FN, COL-III, and COL-I in stromal cells of MSCs group, MSCs + Nalm-6/RS4;11 cells group, TGF-β conditioned MSCs + Nalm-6/RS4;11 cells group, TGF-β conditioned MSCs + Nalm-6/RS4;11 cells + AMD3100 (20 μM) group and TGF-β conditioned MSCs + Nalm-6/RS4;11 cells + AMD3100 (50 μM) group after culturing for 24 h (400×, scale bars, 20 μm).

Previous studies have reported that increased synthesis of extracellular matrix is a significant factor leading to enhanced cell adhesion, and extensive matrix synthesis in the TME is essential for the establishment of a tumor microenvironment that can be infiltrated (Erdogan et al., 2017). Accordingly, in the present study, the expression of matrix protein in MSCs with CAF-like phenotype was observed. First, the basic expression levels of FN, COL-I, and COL-III were detected in MSCs and TGF-β conditioned MSCs. The results revealed that the expression levels of FN, COL-I, and COL-III in TGF-β conditioned MSCs were significantly increased compared with those in MSCs (*P* < 0.05; Figures 5A,B). After MSCs and TGF-β conditioned MSCs were co-cultured with leukemia cells, respectively, the expression levels of the aforementioned matrix protein were further observed. AMD3100 was added to block the interaction between TGF-β conditioned MSCs and ALL cells. Western blot results revealed that, compared with MSCs in the co-culture system, TGF-β conditioned MSCs in the co-culture system had a significantly higher expression of FN, but there was no obvious difference in the expression of COL-I and COL-III between said two groups. After AMD3100 treating, the expression levels of these three matrix proteins in stromal cells were decreased to a certain extent (*P* < 0.05; Figure 5C). Further, the expression of these matrix proteins was also observed by IF, with the results revealing that the expression of FN, COL-III, and COL-I in stromal cells in the TGF-β conditioned MSCs co-culture system had a tendency to increase compared with that of MSCs co-culture system. Yet, the increased expression of matrix proteins did not appear to be weakened by the addition of AMD3100 (Figures 5D,E). Suggesting that AMD3100 might be able to reduce the further secretion of matrix proteins in MSCs with CAF-like phenotype, but the matrix proteins that had increased in MSCs with CAF-like phenotype before treatment could not be well reversed.

### AMD3100 Reduces the Expression of Integrin α5β1 Receptors on the Surface of Leukemia Cells

**FIGURE 6:**
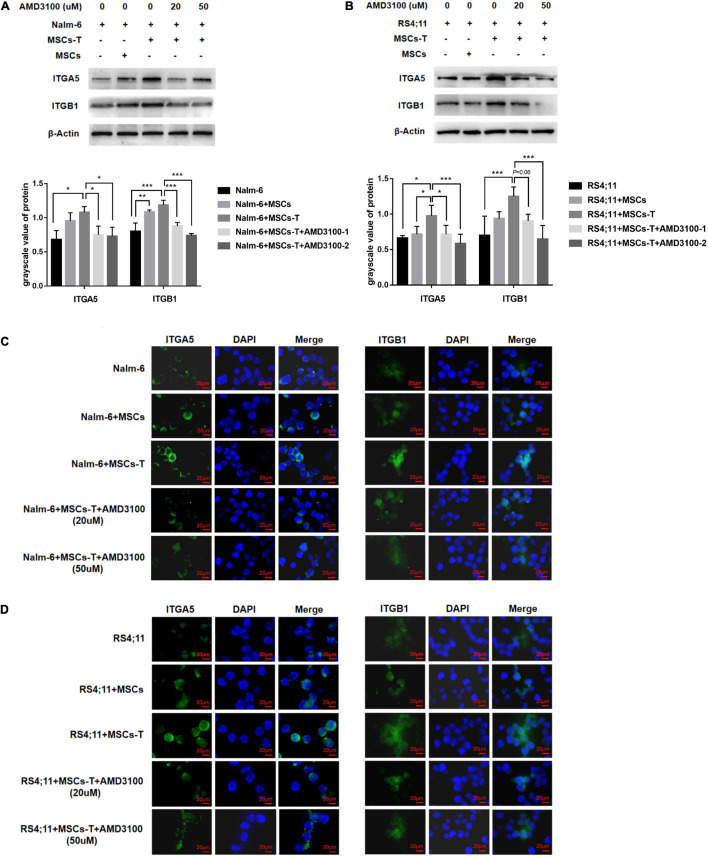
AMD3100 reduced the expression of integrin α5β1 receptors on the surface of leukemia cells. **(A,B)** The expression of ITGA5 and ITGB1 in the Nalm-6/RS4;11 cells from individual culture and co-culture with MSCs, TGF-β conditioned MSCs, TGF-β conditioned MSCs + AMD3100-1 (20 μM) and TGF-β conditioned MSCs + AMD3100-2 (50 μM) for 24 h by western blot. Each value indicates the mean ± SD of three or more independent experiments. In each independent repeated experiment, MSCs was isolated and cultured from a B-ALL patient, and stimulated with rhTGF-β to obtain the CAF-like phenotype, then the latter were compared with the untreated MSCs from the same patient to ensure that the experiment was comparable. *P < 0.05, **P < 0.01, ***P < 0.001. **(C,D)** The expression of ITGA5 and ITGB1 in the above five group by IF (1000×, scale bars, 20 μm).

On the basis of the aforementioned results, an investigation was conducted into whether AMD3100 could prevent the interaction between MSCs with CAF-like phenotype and ALL cells by stimulating certain changes in leukemia cells in the co-culture system. Among the aforementioned extracellular matrix proteins, FN in stromal cells in the TGF-β conditioned MSCs co-culture system was significantly increased, which can bind to integrin receptors on the cell surface to change the adhesion properties of cells and is a significant factor in tumor infiltration (Miyazaki et al., 2020). An assumption was made that the increased secretion of FN in TGF-β conditioned MSCs could mediate the interaction between leukemia cells and TGF-β conditioned MSCs by binding to the main receptor thereof, integrin α5β1, on leukemia cells. Western blot results revealed that the ITGA5 and ITGB1 in leukemia cells in the co-culture system of TGF-β conditioned MSCs group were significantly higher than those in the ALL cells culture alone group (*P* < 0.05; Figures 6A,B), but had no obvious difference compared with the group of co-culture system of MSCs and ALL cells, aside from ITGA5 in leukemia cells in the co-culture system of MSCs and RS4;11 cells (Figures 6A,B). Subsequently, AMD3100 was added to the co-culture system, which weakened the expression of ITGA5 and ITGB1 in leukemia cells (*P* < 0.05; Figures 6A,B). Further, IF was used to detect the expression changes of ITGA5 and ITGB1 in leukemia cells in the aforementioned groups. Results revealed that both ITGA5 and ITGB1 were expressed on the membrane of leukemia cells, and the expression levels of ITGA5 and ITGB1 in leukemia cells in the co-culture system of TGF-β conditioned MSCs exhibited a higher trend, after AMD3100 was added into the co-culture system, the expression levels of ITGA5 and ITGB1 in leukemia cells seemed to decrease (Figures 6C,D). On the basis of such results, an assumption was made that AMD3100 might weaken the interaction between ALL cells and MSCs with CAF-like phenotype by reducing the expression of integrin α5β1 on the surface of leukemia cells.

### Down-Regulating the ITGB1 in Leukemia Cells Could Weaken the Interaction Between Leukemia Cells and Mesenchymal Stem Cells With Cancer-Associated Fibroblast-Like Phenotype

**FIGURE 7:**
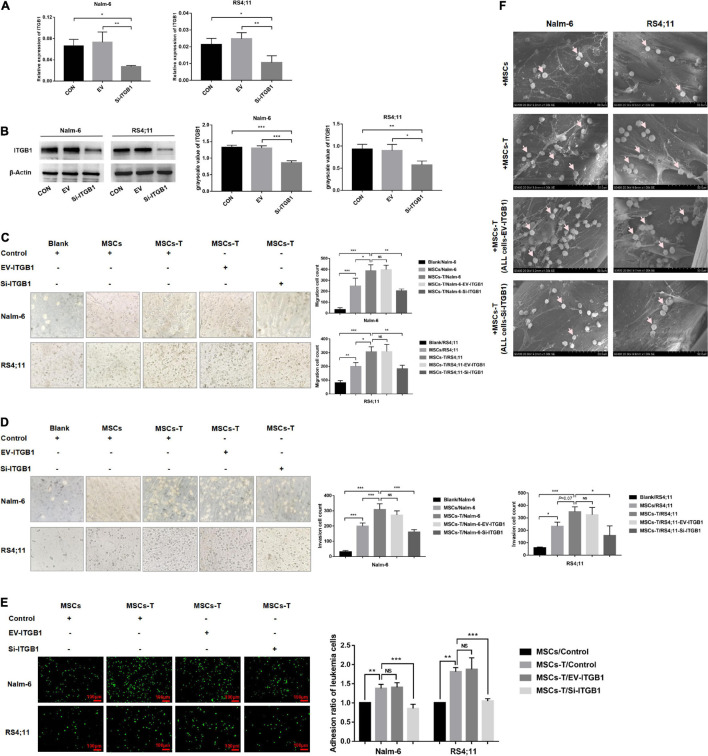
Down-regulating the ITGB1 in leukemia cells could effectively weaken the interaction between leukemia cells and MSCs with CAF-like phenotype. **(A,B)** The mRNA and protein expression levels of ITGB1 in Nalm-6 and RS4;11 cell lines were measured by RT-PCR and western blot after transfection with EV and si-ITGB1 recombinant lentiviral. **(C,D)** Transwell chamber test to detect the number of Nalm-6 and RS4;11 cells migration and invasion in blank, MSCs, TGF-β conditioned MSCs, TGF-β conditioned MSCs/EV-ITGB1 and TGF-β conditioned MSCs/Si-ITGB1 group after 24 h of cells incubation. **(E)** Fluorescence quantitative method detected the adhesion of leukemia cells to stromal cells when Nalm-6/RS4;11 cells were co-cultured with MSCs, TGF-β conditioned MSCs, TGF-β conditioned MSCs/EV-ITGB1 and TGF-β conditioned MSCs/Si-ITGB1 group for 24 h (100×, scale bars, 100 μm). **(F)** The adhesion of leukemia cells to stromal cells when Nalm-6/RS4;11 cells were co-cultured with MSCs, TGF-β conditioned MSCs, TGF-β conditioned MSCs/EV-ITGB1 and TGF-β conditioned MSCs/Si-ITGB1 in a 3D scaffold for 24 h (1000×). Each value indicates the mean ± SD of three or more independent experiments. In each independent repeated experiment, MSCs was isolated and cultured from a B-ALL patient, and stimulated with rhTGF-β to obtain the CAF-like phenotype, then the latter were compared with the untreated MSCs from the same patient to ensure that the experiment was comparable. *P < 0.05, **P < 0.01, ***P < 0.001.

Since ITGB1 is the main component that mediates the adhesion between cells and extracellular matrix components, to further verify the inference of the aforementioned results, a lentiviral transfection reagent was used to down-regulate the expression of ITGB1 in leukemia cells. After down-regulating ITGB1, the successful down-regulation of ITGB1 was verified through RT-PCR and Western blot methods (Figures 7A,B), and the cells were used for subsequent experimental verification. The migration and invasion of controlled leukemia cells, the leukemia cells of the EV group, and the leukemia cells of silent (Si)-ITGB1 were detected. The results revealed that, compared with the leukemia cells in the control group and the EV group co-culturing with TGF-β conditioned MSCs, the migration and invasion of the leukemia cells in the co-culture system of Nalm-6/RS4;11-Si-ITGB1 and TGF-β conditioned MSCs group were significantly weakened (*P* < 0.05; Figures 7C,D). The adhesion of leukemia cells between the groups were detected by using the fluorescence quantitative analysis. As shown in Figure 7E, TGF-β conditioned MSCs significantly promoted the adhesion ability of Nalm-6/RS4;11 cells when compared with the MSCs co-culture group (*P* < 0.05), and Si-ITGB1 in Nalm-6/RS4;11 cells could weaken the promotion effect of TGF-β conditioned MSCs (*P* < 0.05). Further verification by 3D co-culture system showed that the results seemed to be consistent with the fluorescence quantitative method (Figure 7F), suggesting that down-regulating the ITGB1 in leukemia cells might effectively reduce the interaction between MSCs with CAF-like phenotype and ALL cells.

### Up-Regulating the ITGB1 in Leukemia Cells Could Partially Reverse the Inhibition Effect of AMD3100 on the Interaction Between Leukemia Cells and Mesenchymal Stem Cells With Cancer-Associated Fibroblast-Like Phenotype

**FIGURE 8:**
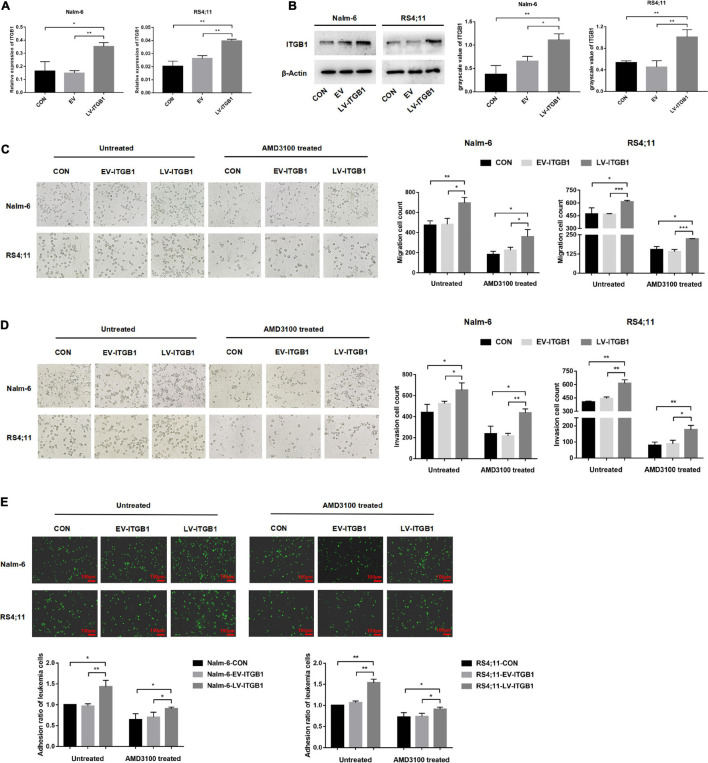
Up-regulating the ITGB1 in leukemia cells could partially reverse the inhibition effect of AMD3100 on the interaction between leukemia cells and MSCs with CAF-like phenotype. **(A,B)** The mRNA and protein expression levels of ITGB1 in Nalm-6 and RS4;11 cell lines were measured by RT-PCR and western blot after transfection with EV and LV-ITGB1 recombinant lentiviral. **(C,D)** Transwell chamber test to detect the effect of TGF-β conditioned MSCs on the migration and invasion of Nalm-6 and RS4;11 cells in the control group, the EV group, and the LV-ITGB1 group, as well as the changes in the migration and invasion of the above groups after AMD3100 (50 μM) treating (cultured for 24 h). **(E)** Fluorescence quantitative method detected the adhesion of leukemia cells to stromal cells when TGF-β conditioned MSCs were co-cultured with control group of leukemia cells, EV group of leukemia cells and LV-ITGB1 group of leukemia cells for 24 h, respectively. As well as the changes in the adhesion of the above groups after AMD3100 (50 μM) treating (100×, scale bars, 100 μm). Each value indicates the mean ± SD of three independent experiments. *P < 0.05, **P < 0.01, ***P < 0.001.

In the following experiments, a lentiviral transfection reagent was used to up-regulate the expression of ITGB1 in leukemia cells to further verify the inference of the above results. The successful up-regulation of ITGB1 was verified through RT-PCR and Western blot methods (Figures 8A,B), and the effects of TGF-β conditioned MSCs on the migration, invasion and adhesion of leukemia cells in the control group, the EV group, and the LV-ITGB1 group were detected, AMD3100 was used to interfere with the interaction between leukemia cells and TGF-β conditioned MSCs. The transwell chamber test revealed that compared with the leukemia cells in the control group and the EV group co-culturing with TGF-β conditioned MSCs, the migration and invasion of the leukemia cells in the co-culture system of Nalm-6/RS4;11-LV-ITGB1 and TGF-β conditioned MSCs group were significantly enhanced (*P* < 0.05; Figures 8C,D). After AMD3100 treating, the migration and invasion numbers of leukemia cells in the control group and the EV group were obviously decreased, but up-regulation of ITGB1 in leukemia cells could partially reverse the inhibition effect of AMD3100 (*P* < 0.05; Figures 8C,D). Further, the adhesion of leukemia cells between the groups were detected by using the fluorescence quantitative analysis. As shown in Figure 8E, compared with the control group and the EV group, the adhesion of leukemia cells in the LV-ITGB1 group increased obviously (*P* < 0.05). In addition, up-regulation of ITGB1 partially weaken the inhibitory effect of AMD3100 on the adhesion of leukemia cells under AMD3100 treatment (Figure 8E, *P* < 0.05). Suggesting that up-regulating the ITGB1 in leukemia cells could enhance the promotion effect of MSCs with CAF-like phenotype on the migration, invasion and adhesion of leukemia cells, and partially reverse the interference of AMD3100 on the interaction between leukemia cells and MSCs with CAF-like phenotype.

## Discussion

The proliferation, migration and invasion of leukemia cells are significant factors leading to the progression and poor prognosis of leukemia. Studies have indicated that cancer cells are prone to drug resistance owing to the genome instability thereof, non-tumor cells in the TME have genetically more stable characteristics and are more vulnerable, and targeting the TME might have significant therapeutic advantages over directly targeting cancer cells (Xiao and Yu, 2021). BM-MSCs as the stromal cells in the TME, they could obtain CAF-like phenotype under certain conditions to further promote the progression of leukemia. But the *in vivo* experiments of the MSCs with CAF-like phenotype on the progression of B-ALL have not been verified, and the mechanism by which MSCs with CAF-like phenotype promotes the migration and invasion of B-ALL cells remains unclear and has not been disclosed in other studies. So the present study launched further exploration, so as to provide a strong experimental basis for finding potential treatments.

In a variety of tumors, CAFs have been considered as a key matrix component to promote tumorigenesis and progression (Alexander and Cukierman, 2020; Joshi et al., 2021). Data from previous studies by the present authors (Pan et al., 2020) revealed that BM-MSCs could obtain CAF-like phenotypes with high expression of α-SMA and FAP in the B-ALL microenvironment. Since α-SMA and FAP could not fully characterize the characteristics of CAFs in the present study, the MSCs were referred to as MSCs with CAF-like phenotype. Previous reports have shown that MSCs and CAFs have many similarities, both being adherent and exhibiting spindle-shaped growth, compared with MSCs, CAFs have higher expression levels of surface markers and cytokines (Paunescu et al., 2011). In the present study, MSCs with CAF-like phenotype expressed all the cell surface markers commonly used to mark MSCs, but the adipogenic differentiation function was weakened. And the expression levels of the biomarkers of MSCs and MSCs with CAF-like phenotype were confirmed to be different in a previous study by the present authors (Pan et al., 2020), indicating that there is a certain difference there between.

In the vivo experiments, MSCs with CAF-like phenotype promoted tumor formation and stimulated tumor growth in mice, which is consistent with previously published studies on solid tumors (Teng et al., 2016). Studies have considered a number of possible mechanisms through which CAFs promote the growth and proliferation of tumor cells, including paracrine cytokines (Li et al., 2019; Zhang J. et al., 2020), producing extracellular matrix proteins (Erdogan et al., 2017), promoting extracellular matrix remodeling (Neri et al., 2016; Stylianou et al., 2019) and cell–cell interactions (Miyazaki et al., 2020; Conti et al., 2021). SDF-1/CXCR4 is a key signal axis that mediates the communication between tumor cells and stromal cells. Izumi et al. (2016) reported that CAFs could participate in tumor invasion and metastasis by activating the SDF-1/CXCR4 signaling pathway. Teng et al. (2016) also found that CAFs promoted the progression of endometrial cancer through the SDF-1α/CXCR4 axis. In the present study, the CXCR4 had a trend of increasing expression in the MSCs with CAF-like phenotype and ALL cells co-injection group, and high expression of CXCR4 was observed in ALL primary cells and ALL cell lines to varying degrees. Further, the ligand SDF-1 had the highest expression in MSCs with CAF-like phenotype, suggesting that SDF-1 derived from MSCs with CAF-like phenotype could be a key factor in stimulating the activation of the SDF-1/CXCR4 signal axis in the leukemia microenvironment, the latter further promote the interaction of MSCs with CAF-like phenotype and ALL cells. Not enough is that the present study did not further confirm the role of CXCR4 in tumor progression and whether the application of CXCR4 inhibitor could effectively delay tumor development through *in vivo* experiments. Previous studies have reported that CXCR4 is a key factor that promotes the growth, proliferation and infiltrating of leukemia cells (Crazzolara et al., 2001; Tavor et al., 2004; Liu et al., 2011; de Lourdes Perim et al., 2015). In the *in vivo* experiments, CXCR4 has been confirmed as a key component to promote the bone marrow homing of leukemia cells (Juarez et al., 2009). The combination of CXCR4 inhibitors and chemotherapeutics could achieve better remissions in leukemia cells and NOD/SCID mice model of ALL (Welschinger et al., 2013; Jin et al., 2018; Usmani et al., 2019). Suggesting that CXCR4 might play an important role in the progression of ALL.

AMD3100, a specific CXCR4 inhibitor, has been demonstrated to effectively prevent the transduction of the SDF-1/CXCR4 signal (van den Berk et al., 2014; Tan et al., 2020). Previous studies have reported that the AMD3100 could enhance the sensitivity of leukemia cells to chemotherapeutics when combined with chemotherapy drugs, and the likely mechanism was to mobilize leukemia cells in the bone marrow to release into peripheral blood, thereby promoting the killing of leukemia cells by chemotherapeutics (Juarez et al., 2007; Dillmann et al., 2009; Welschinger et al., 2013; Wang et al., 2020). Recently, AMD3100 has entered phase I clinical trials in patients with acute leukemia and myelodysplastic syndrome (Cooper et al., 2017). In this study, findings were made that AMD3100 alone had no obvious pro-apoptotic effect on leukemia cells, but could effectively reduce the promotion effects of MSCs with CAF-like phenotype on the migration and invasion of ALL cells through *in vitro* experiments. AMD3100 could also inhibit the secretion of MMP-9 in MSCs with CAF-like phenotype and block the activation of the PI3K/AKT signaling pathway in ALL cells. MMP-9 has been proven to promote the invasion of tumor cells into surrounding tissues, and the metastasis to distant tissues in a variety of tumors (Hwang et al., 2021; Yin et al., 2021). In addition, the PI3K/AKT signaling pathway has been found to be a significant factor in regulating tumor cell migration, invasion and survival (Wang et al., 2018; Sanchez et al., 2019). An assumption was made that AMD3100 may have the potential to inhibit the migration and invasion of ALL cells by interfering with the interaction between MSCs with CAF-like phenotype and leukemia cells. For the ERK signaling pathway, it has been shown to be a signaling pathway that promotes the growth and proliferation of leukemia cells in previous reports (Chang et al., 2020; Liu et al., 2020; Song et al., 2020). In the present study, the phosphorylation level of the ERK signaling pathway in the co-culture system tended to increase, but there was no statistical significance in each group, and the expression thereof was not significantly reduced after receiving AMD3100 treatment, suggesting that the phosphorylation level of the ERK signaling pathway may not be limited to the influence of the SDF-1/CXCR4 axis.

In the following experiments, a 3D culture model was used, with the findings being that MSCs with CAF-like phenotype could attract more leukemia cells to adhere to the surface thereof than MSCs, and some leukemia cells could hide in the stromal fibers, but the adherent leukemia cells was significantly reduced after adding AMD3100. Up to now, there has been no information on whether B-ALL cells can be made more sensitive to therapeutic agents by using AMD3100 to disrupt the adhesion thereof to MSCs with CAF-like phenotype. However, in research on AML, data by Bray et al. (2017) revealed that, compared with 2D cultured cells, 3D cultured cells were more resistant to chemotherapeutics, and the application of AMD3100 could induce AML cells to mobilize from the blood vessel wall. The results seem to be consistent with those of the present study.

Studies have shown that CAFs could change the structure and physical properties of the extracellular matrix (ECM), thereby affecting cell migration, invasion and growth (Erdogan et al., 2017). In previous reports on ALL cells, the existence of abnormally proliferating fibrous structures were found in bone marrow, which formed specific microniches to protect the remaining leukemia cells, leading to recurrence of the disease (Duan et al., 2014). In the present study, the ability of MSCs with CAF-like phenotype to secrete matrix proteins was found to significantly increase, suggesting that the increased secretion of matrix fibrin from MSCs with CAF-like phenotype might be a significant cause of abnormal proliferation of fibrous structures in the microenvironment, and may provide a path and environment for tumor cells to invade and metastasize more easily.

Chen et al. (2019) reported that the elimination of CXCR4 signaling in CAFs could reduce the level of fibrosis and αSMA^+^ cells, leading to the normalization of the vasculature. However, the present results revealed that AMD3100 reduced the further expression of matrix proteins in MSCs with CAF-like phenotype, but the increased matrix fibers produced by MSCs with CAF-like phenotype could not be effectively reversed. Thus, after shifting focus to leukemia cells, notable findings were made that AMD3100 could reduce the expression of FN receptor, integrin α5β1, in leukemia cells. The latter was considered to be a significant mechanoreceptor for the interaction between tumor cells and matrix components (Jang and Beningo, 2019). Down-regulating the ITGB1 on leukemia cells could significantly reduce the adhesion, migration and invasion effects of MSCs with CAF-like phenotype on leukemia cells. In turn, up-regulating ITGB1 in leukemia cells could enhance the interaction between leukemia cells and MSCs with CAF-like phenotype, and partially reverse the interference of AMD3100 on the interaction between leukemia cells and MSCs with CAF-like phenotype, indicating that targeting ITGB1 may be an effective treatment method to reduce the disease progression and extramedullary infiltration of leukemia cells. Such findings have also been reported in a previous study on solid tumors (Li et al., 2018). In summary, targeted malignant cell transport will provide a new treatment method for B-ALL. By inhibiting adhesion and inducing mobilization, the ability of tumor cells to interact with MSCs with CAF-like phenotype will be altered, thereby increasing the sensitivity to therapeutic drugs.

**FIGURE 9:**
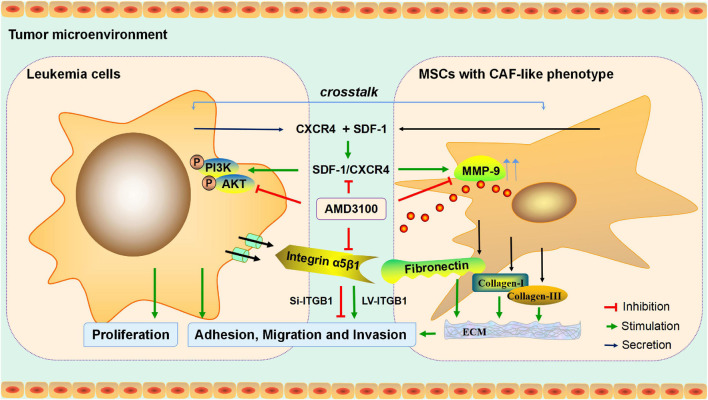
Mechanism diagram. MSCs with CAF-like phenotype stimulate SDF-1/CXCR4 axis to enhance the growth and invasion of B-ALL cells through cell-to-cell communication. AMD3100 reduces the secretion of MMP-9 in MSCs with CAF-like phenotype and the phosphorylation of PI3K/AKT signaling pathway on ALL cells by blocking the SDF-1/CXCR4 axis, and weakens the expression of integrin receptor α5β1 on leukemia cells to reduce the interaction between the integrin α5β1 and the increased secretion of fibronectin in MSCs with CAF-like phenotype, thereby reducing the interaction between MSCs with CAF-like phenotype and ALL cells. Regulation of ITGB1 in leukemia cells could effectively interfere with the promoting effect of MSCs with CAF-like phenotype on the adhesion, migration and invasion of leukemia cells.

To conclude, in the present study, an investigation was conducted into the interaction between MSCs with CAF-like phenotype and leukemia cells in promoting the progression of B-ALL, and the potential molecular mechanism was determined (Figure 9), which may provide an experimental basis for the further exploration of the potential mechanism of proliferation and invasion of leukemia cells, and may provide a reference for the treatment of inhibiting extramedullary infiltration of B-ALL.

## Data Availability Statement

The original contributions presented in the study are included in the article/Supplementary Material, further inquiries can be directed to the corresponding author/s.

## Ethics Statement

The studies involving human participants were reviewed and approved by the Ethics Committee of the Affiliated Hospital of Guizhou Medical University. The patients/participants provided their written informed consent to participate in this study. The animal study was reviewed and approved by the Animal Care Welfare Committee of Guizhou Medical University.

## Author Contributions

CP designed the study, performed the research, and prepared the manuscript. QF helped design the research and assisted with manuscript preparation. PL assisted with data analysis and performed the research. DM assisted with research design. SC, LZ, and QC provided the clinical samples and healthy donors. TH helped to performing the research. JW designed the research and provided final approval of the manuscript. All the authors read and approved the final manuscript.

## Conflict of Interest

The authors declare that the research was conducted in the absence of any commercial or financial relationships that could be construed as a potential conflict of interest.

## Publisher’s Note

All claims expressed in this article are solely those of the authors and do not necessarily represent those of their affiliated organizations, or those of the publisher, the editors and the reviewers. Any product that may be evaluated in this article, or claim that may be made by its manufacturer, is not guaranteed or endorsed by the publisher.

## Supplementary Material

Supplementary Figure S1 The immunophenotype, osteogenic and adipogenic differentiation functions of MSCs with CAF-like phenotype. **(A)** Immunophenotype identification of MSCs with CAF-like phenotype by flow cytometry. **(B)** Representative images of Oil Red O-stained MSCs and TGF-β conditioned MSCs following 15 days of adipogenic induction (200×). **(C)** Representative images of Alizarin Red stained MSCs and TGF-β conditioned MSCs following 21 days of osteogenic induction (200×).

Supplementary Figure S2 AMD3100 was been used with an appropriate dosage to block the SDF-1/CXCR4 signal axis. **(A)** Nalm-6 and RS4; 11 cell lines were treated with AMD3100 at the concentrations of 0, 10, 20, 30, 40, and 50 μM for 24 h, then western blot was used to detect the expression of CXCR4. **(B)** The Nalm-6 and RS4;11 cell lines were treated with AMD3100 at the concentrations of 0, 10, 20, 30, 40, and 50 μM for 24 h, and then flow cytometry was used to detect the apoptosis of Nalm-6 and RS4;11 cell lines. **(C,D)** Statistical analysis of the apoptosis of Nalm-6 and RS4;11 cell lines in the above six groups. Each value indicates the mean ± SD. ^∗^*P* < 0.05, ^∗∗^*P* < 0.01, ^∗∗∗^*P* < 0.001. NS, no significant statistical difference.

Supplementary Figure S3 Up-regulation of CXCR4 in leukemia cells could enhance the promotion effect of MSCs with CAF-like phenotype on the migration and invasion of leukemia cells. **(A,B)** The mRNA and protein expression levels of CXCR4 in Nalm-6 and RS4;11 cell lines were measured by RT-PCR and western blot after transfection with EV and LV-CXCR4 recombinant lentiviral. **(C,D)** Transwell chamber test to detect the number of Nalm-6 and RS4;11 cells migration and invasion in blank, MSCs, TGF-β conditioned MSCs, TGF-β conditioned MSCs/EV-CXCR4 and TGF-β conditioned MSCs/LV-CXCR4 group after 24 h of cells incubation. Each value indicates the mean ± SD of three independent experiments. In each independent repeated experiment, MSCs was isolated and cultured from a B-ALL patient, and stimulated with rhTGF-β to obtain the CAF-like phenotype, then the latter were compared with the untreated MSCs from the same patient to ensure that the experiment was comparable. ^∗^*P* < 0.05, ^∗∗^*P* < 0.01, ^∗∗∗^*P* < 0.001.
