# Supplementary figures and images for: Mesenchymal Stem Cells With Cancer-Associated Fibroblast-Like Phenotype Stimulate SDF-1/CXCR4 Axis to Enhance the Growth and Invasion of B-Cell Acute Lymphoblastic Leukemia Cells Through Cell-to-Cell Communication
Source: Front Cell Dev Biol. 2021 Oct 18;9:708513. doi: 10.3389/fcell.2021.708513 (PMC8558501; doi:10.3389/fcell.2021.708513)

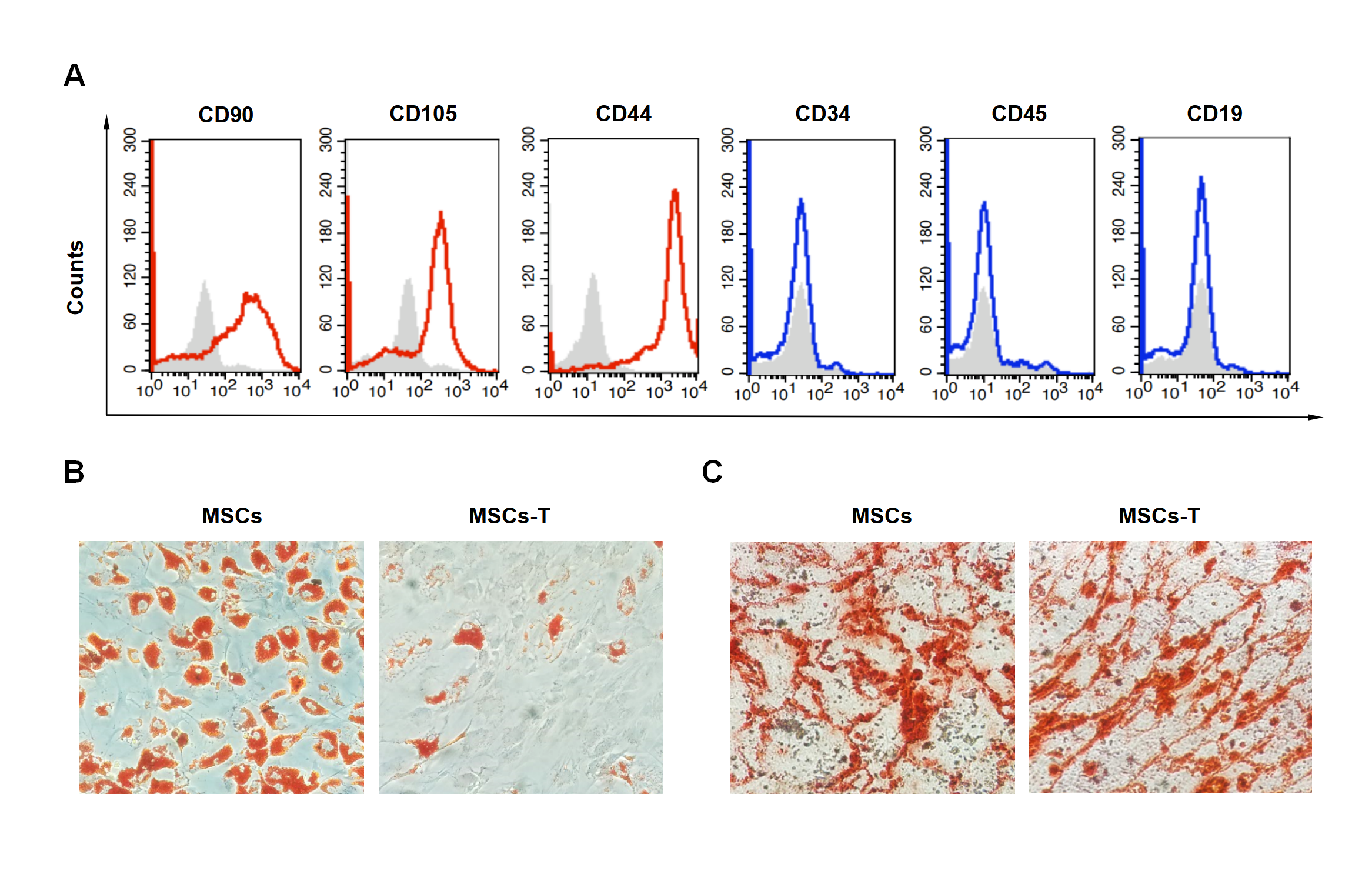

Supplement: Supplementary Figure S1 — The immunophenotype, osteogenic and adipogenic differentiation functions of MSCs with CAF-like phenotype. (A) Immunophenotype identification of MSCs with CAF-like phenotype by flow cytometry. (B) Representative images of Oil Red O-stained MSCs and TGF-β conditioned MSCs following 15 days of adipogenic induction (200×). (C) Representative images of Alizarin Red stained MSCs and TGF-β conditioned MSCs following 21 days of osteogenic induction (200×). [file Data_Sheet_1.zip › Figure S1.tif]

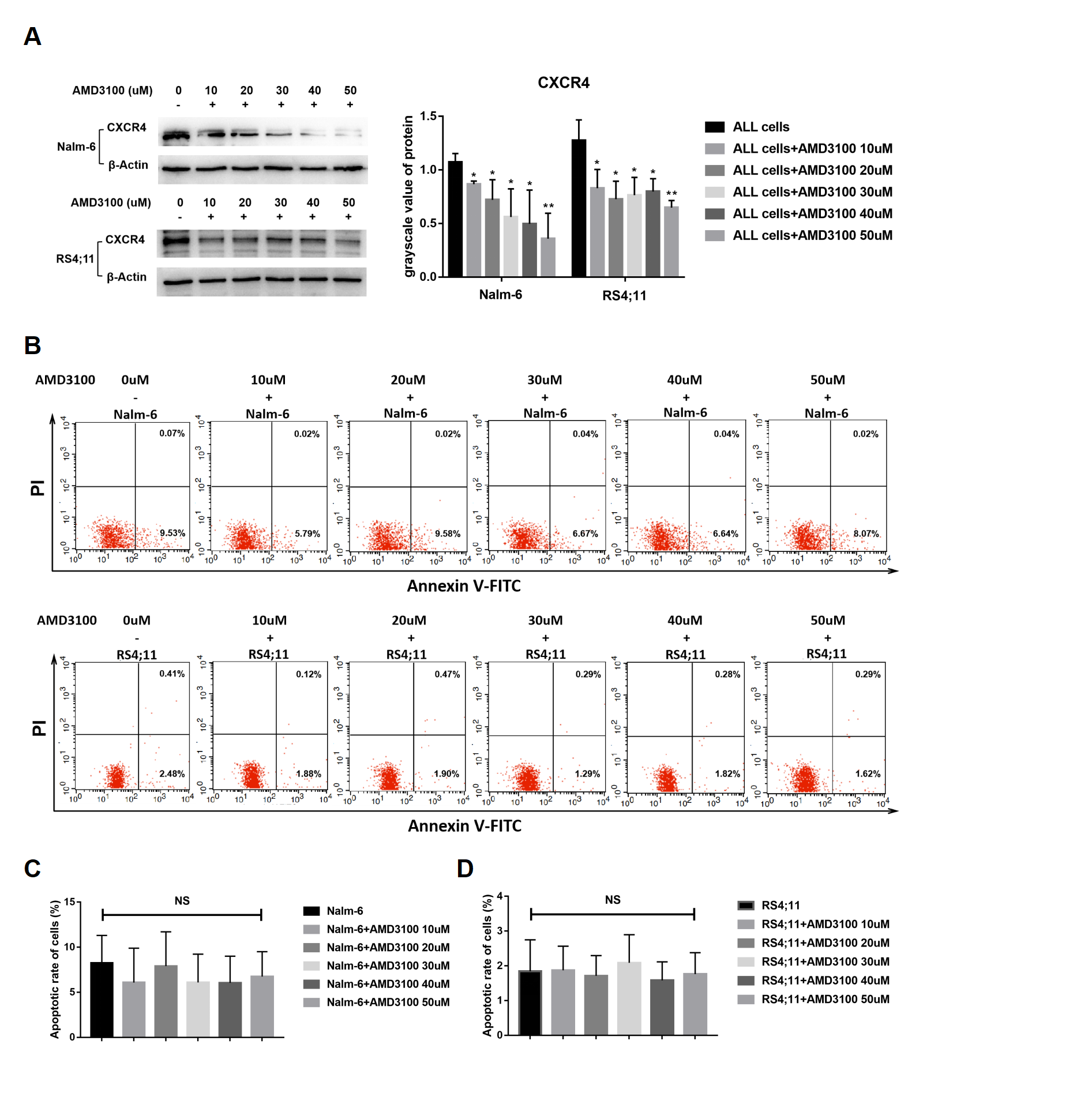

Supplement: Supplementary Figure S1 — The immunophenotype, osteogenic and adipogenic differentiation functions of MSCs with CAF-like phenotype. (A) Immunophenotype identification of MSCs with CAF-like phenotype by flow cytometry. (B) Representative images of Oil Red O-stained MSCs and TGF-β conditioned MSCs following 15 days of adipogenic induction (200×). (C) Representative images of Alizarin Red stained MSCs and TGF-β conditioned MSCs following 21 days of osteogenic induction (200×). [file Data_Sheet_1.zip › Figure S2.tif]

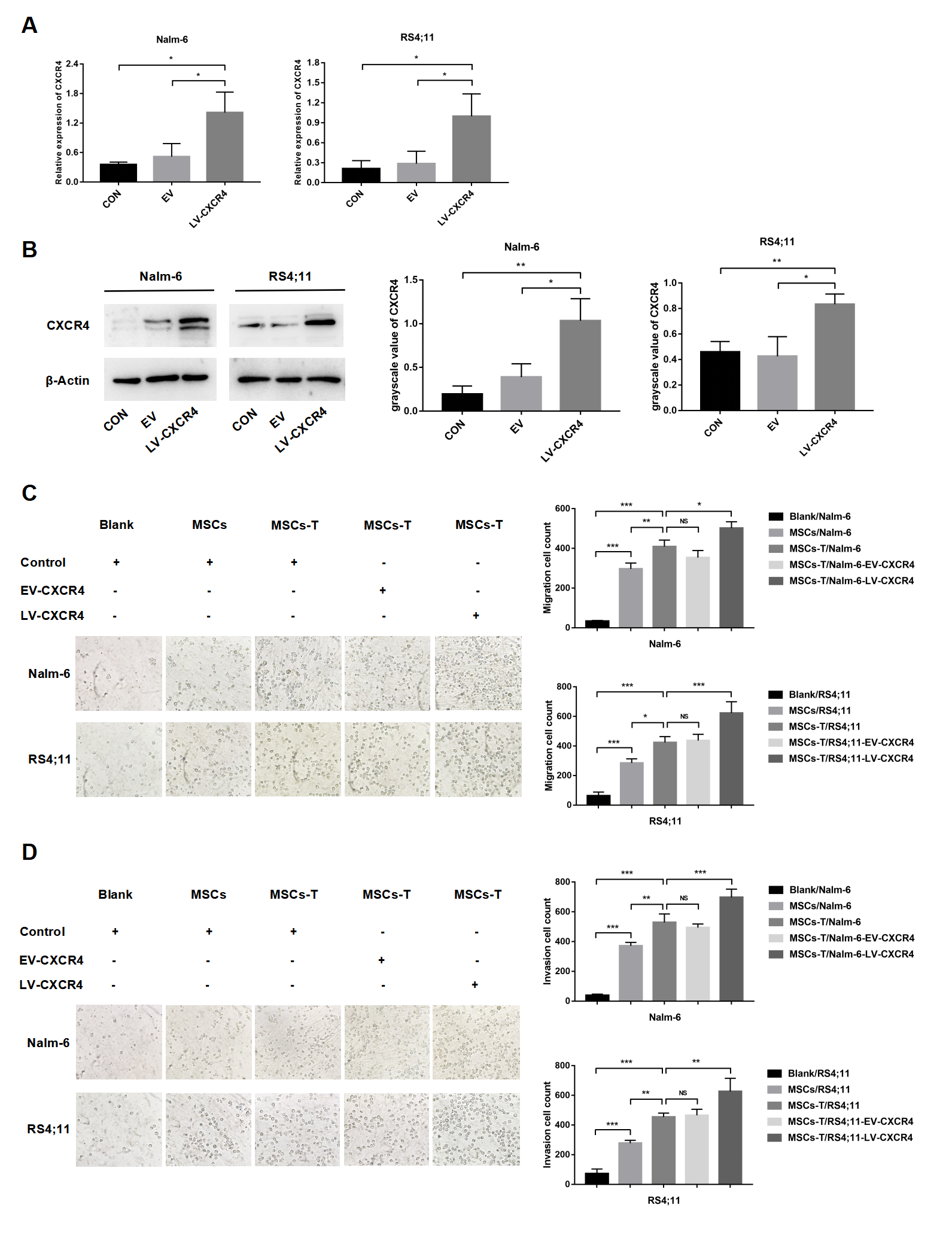

Supplement: Supplementary Figure S1 — The immunophenotype, osteogenic and adipogenic differentiation functions of MSCs with CAF-like phenotype. (A) Immunophenotype identification of MSCs with CAF-like phenotype by flow cytometry. (B) Representative images of Oil Red O-stained MSCs and TGF-β conditioned MSCs following 15 days of adipogenic induction (200×). (C) Representative images of Alizarin Red stained MSCs and TGF-β conditioned MSCs following 21 days of osteogenic induction (200×). [file Data_Sheet_1.zip › Figure S3.tif]
